# *Legionella pneumophila* as Cause of Severe Community-Acquired Pneumonia, China

**DOI:** 10.3201/eid2601.190655

**Published:** 2020-01

**Authors:** Huahua Yi, Jie Fang, Jingwen Huang, Bing Liu, Jieming Qu, Min Zhou

**Affiliations:** Ruijin Hospital, Shanghai, China

**Keywords:** community-acquired pneumonia, pneumonia, *Legionella pneumophila*, next-generation sequencing, bacteria, pneumonia, respiratory infections, China

## Abstract

We report a case of community-acquired pneumonia in a patient in China. We verified *Legionella pneumophila* infection through next-generation sequencing of blood, sputum, and pleural effusion samples. Our results show the usefulness of next-generation sequencing and of testing different samples early in the course of illness to identify this bacterium.

Community-acquired pneumonia (CAP) can lead to high mortality rates worldwide (*1*). Possible causes are *Streptococcus pneumoniae, Haemophilus influenzae*, *Mycoplasma chlamydia,* and *Legionella* spp. bacteria, as well as various respiratory viruses; although identifying pathogens in a timely manner is critical, it is not done in most cases. Next-generation sequencing (NGS) has emerged as a high-throughput method of pathogen identification and is superior to current microbiologic diagnostic measures for identifying hard-to-culture pathogens. We report a case of CAP in China caused by *Legionella pneumophila*, determined through NGS of sputum, blood, and pleural effusion samples.

The patient was a 65-year-old woman with a history of breast cancer who sought care for fever on December 1, 2018, in a hospital in her community (Appendix 1); her fever was accompanied by vomiting. Emergency room staff diagnosed gastroenteritis and prescribed ceftriaxone. The next day, she experienced frequent urination and urinary pain followed by diarrhea and returned to the hospital. A computed tomography (CT) scan of her abdomen showed gallbladder calculi and a low-density node in the right lobe of the liver. Treatment did not resolve fever and diarrhea. On the fifth day, the patient experienced respiratory symptoms including cough and purulent sputum and was admitted to the gastroenterology department in the same hospital. Over the next 2 days, respiratory symptoms worsened, and she was transferred to the infectious disease department of Ruijin Hospital in Shanghai. 

At admission, PCR and serologic testing we performed, as well as bacterial culture from blood and sputum samples. Chest CT results showed patchy consolidation in the lower lobe of the left lung (Figure, panels A and B). The patient was transferred to respiratory intensive care. A respiratory clinician, suspecting *L. pneumophila* as the cause, prescribed the quinolone moxifloxacin. We repeated serologic tests for *L. pneumophila* and cultured respiratory samples again. We sent samples of blood, sputum, and pleural effusion to BGI Group (Shenzhen, China) for NGS, and urine to Shanghai East Hospital for *L. pneumophila* antigen testing. Serum antibody and urine antigen tests were negative. but NGS indicated *L. pneumophila* in blood, sputum, and pleural effusion. For blood, the coverage rate of *L. pneumophila* was 3.2% with 1,136 raw reads; it was 3.8% with 1,353 raw reads for sputum and 8% with 2,867 raw reads for pleural effusion (Appendix 2 Tables 1−3). Under moxifloxacin treatment, the patient’s symptoms disappeared in 1 week. We conducted another NGS for blood without any evidence for *L. pneumophila*. Patchy consolidation in the left lower lung lobe was almost completely absorbed (Figure, panel C).

**Figure:**
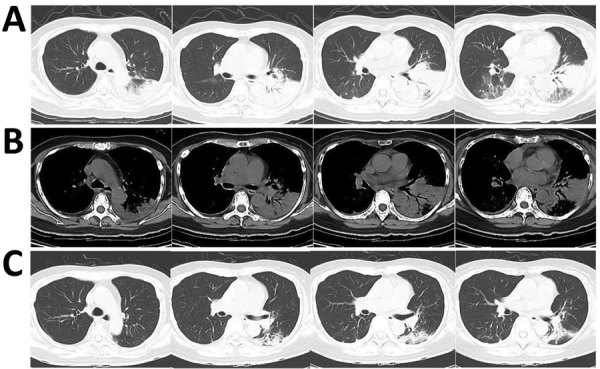
Computed tomography images of the lungs of a 65-year-old woman with community-acquired pneumonia caused by *Legionella pneumophila* bacteria, China, showing absorption of infusion in left lobes after effective treatment. Lung window (A) and bone window (B) at the beginning of treatment showed consolidation in the left lobes; after 1 week of treatment (C), infusion is mostly absorbed in the left lobes.

We confirmed the presence of *L. pneumophila* bacteria from by PCR for the remaining pleural infusion and sputum of this patient. We did not investigate the source of the *L. pneumophila* bacteria.

*L. pneumophila* bacteria is 1 of the 3 most common pathogens that cause severe CAP and is isolated in 1%–40% of hospital-acquired pneumonia cases (*2*). Extrapulmonary symptoms are always present in *L. pneumophila* infection, which may increase the risk for misdiagnosis early in the infection. Furthermore, culturing *L. pneumophila* requires a specific medium, which makes it difficult to obtain a diagnosis without positive microbiology test results. *L. pneumophila* infections have been reported previously (*3*,*4*) but were confirmed by PCR with target microorganisms and not by NGS. 

BGI Group conducted NGS as described previously (*5*), collecting 3–4 mL blood in ethylenediaminetetraacetic acid tubes, staining in RT for 3–5 minutes, and centrifuging at 1,600 × *g* for 10 min at 4°C. They then collected 0.5–3 mL sputum or pleural effusion sample following standard procedures and, using a 1.5-mL microcentrifuge tube, agitated the sample at 2,800–3,200 rpm for 30 min. They separated 0.3 mL of the sample into a new tube and extracted DNA using the TIANamp Micro DNA Kit (DP316; Tiangen Biotech, http://tiangen.com) according to the manufacturer’s recommendation. DNA libraries were constructed through DNA fragmentation, end repair, adapt ligation, and PCR amplification. 

BGI Group sequenced the qualified libraries by using the BGISEQ-100 platform (*6*). They generated high-quality sequencing data by removing low-quality and short (length <35 bp) reads, then performed computational subtraction of human host sequences mapped to the human reference genome (hg19) using Burrows-Wheeler alignment (*7*). They classified the remaining data by removing low-complexity reads and simultaneously aligning the sequences to microbial genome databases for bacteria, viruses, fungi, and parasites downloaded from the US National Center for Biotechnology Information (ftp://ftp.ncbi.nlm.nih.gov/genomes). Experts in respiratory illness, microbiology, and radiology evaluated patients’ status and interpreted results of NGS testing together, thus identifying possible etiologic agents.

Because turnaround time is short and nonspecific primers can be used, NGS is useful for detecting unculturable pathogens, especially in severely ill patients (*8*). NGS is highly sensitive and expensive, so clinicians must assess the value of NGS for identifying etiologic pathogens. In this case, we diagnosed *L. pneumophila* infection on the basis of 3 kinds of samples that had not been reported previously, which shows the importance of testing multiple samples early in the course of illness to identify the etiologic agent and begin appropriate treatment.

Appendix 1 Timeline of illness in a case of community-acquired pneumonia caused by *Legionella pneumophila* bacteria, China.

Appendix 2 Additional data for investigation of a case of community-acquired pneumonia caused by *Legionella pneumophila* bacteria, China.
