# Supplementary material for: Legionella pneumophila as Cause of Severe Community-Acquired Pneumonia, China
Source: Emerg Infect Dis. 2020 Jan;26(1):160–2. doi: 10.3201/eid2601.190655 (PMC6924908; doi:10.3201/eid2601.190655)
Supplement: Appendix 1 — Timeline of illness in a case of community-acquired pneumonia caused by Legionella pneumophila bacteria, China. [file 19-0655-Techapp-s1.pdf]

Article DOI: https://doi.org/10.3201/eid2601.190655

Legionella pneumophila as a Cause of Severe Community-Acquired Pneumonia, China

Appendix

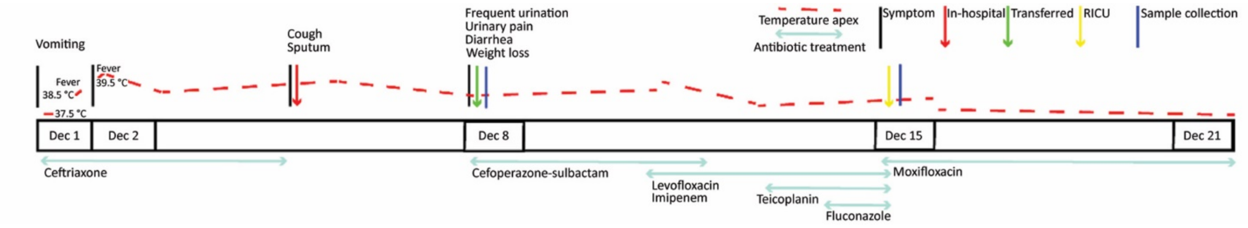

Appendix Figure. Illness and treatment over time of a patient with community-acquired pneumonia caused by *Legionella pneumophila* bacteria, China.
